# Middle East Respiratory Syndrome Vaccine Candidates: Cautious Optimism

**DOI:** 10.3390/v11010074

**Published:** 2019-01-17

**Authors:** Craig Schindewolf, Vineet D. Menachery

**Affiliations:** Department of Microbiology and Immunology, University of Texas Medical Branch, Galveston, 77555 TX, USA; crschind@UTMB.EDU

**Keywords:** Middle East respiratory syndrome coronavirus, severe acute respiratory syndrome coronavirus, coronavirus spike glycoprotein, vaccine platforms, correlates of immunity, animal models

## Abstract

Efforts towards developing a vaccine for Middle East respiratory syndrome coronavirus (MERS-CoV) have yielded promising results. Utilizing a variety of platforms, several vaccine approaches have shown efficacy in animal models and begun to enter clinical trials. In this review, we summarize the current progress towards a MERS-CoV vaccine and highlight potential roadblocks identified from previous attempts to generate coronavirus vaccines.

## 1. Introduction

In recent years, viral zoonotic diseases have caused outbreaks marked by rapid spread and high mortality, including the 2002 emergence of severe acute respiratory syndrome coronavirus (SARS-CoV) [1], the 2009 H1N1 swine flu pandemic [2], and the 2013 Ebola outbreak in West Africa [3]. Such outbreaks are difficult to predict as new strains emerge or reemerge from zoonotic reservoirs [4]. Coronaviruses (CoVs), large positive-stranded RNA viruses of the order *Nidovirales* [5], were considered minor human pathogens, causing cold-like symptoms and occasionally associated with pneumonia and more severe disease [6]. However, the emergence of SARS-CoV in 2002 and Middle East respiratory syndrome coronavirus (MERS-CoV) in 2012, members of βCoV lineages B and C, respectively, marked a shift in our understanding of the pathogenic potential of coronaviruses [7]. As these more virulent viruses are genetically similar to those currently circulating in bats [8,9], CoVs may pose a threat for future zoonoses [10].

Since the emergence of MERS-CoV in Saudi Arabia in 2012, over 2200 confirmed cases have been reported in at least 27 countries, with an overall mortality rate of 35% (https://www.who.int/emergencies/mers-cov). Additionally, more severe disease has been noted in the aged, immunocompromised, and those with chronic health conditions [11]. Camels, which show seropositivity to MERS-CoV in archived sera dating back to 1983 [12], serve as intermediate hosts and are able to spread the virus to humans [13], who may then spread the infection person-to-person [14]. While a range of therapeutics have been explored for CoV disease [15,16,17], a MERS-CoV vaccine remains the most scalable, cost-effective prophylactic measure. Currently, a vaccine for MERS-CoV is not available, although several candidates have been developed using a variety of approaches. Vaccine studies were initially hampered by a lack of small animal models of MERS-CoV disease [18]. While rodents possess homologues for dipeptidyl peptidase 4 (DPP4), the human receptor for MERS-CoV [19], rodent DPP4 homologues are incompatible with MERS-CoV infection [20,21,22]. However, several in vivo approaches have been developed to overcome these barriers and facilitate MERS-CoV vaccine testing in small animal models [23,24,25,26,27].

**Figure 1:**
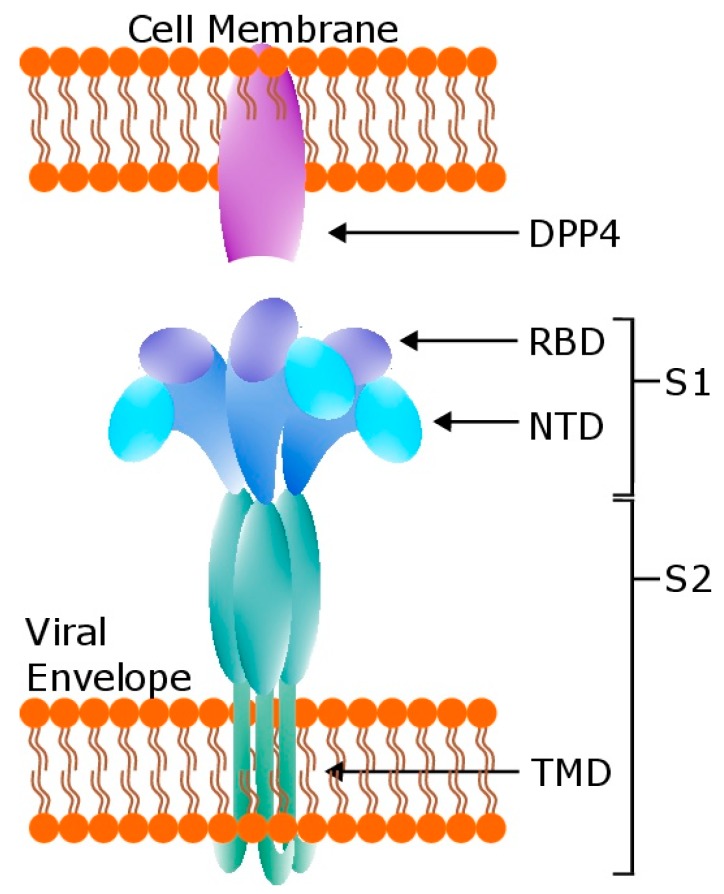
The MERS-CoV spike (S) glycoprotein, a Class I fusion protein and the target of the majority of vaccine candidates, exists naturally in trimer form as shown in this simplified diagram. DPP4: dipeptidyl peptidase 4, the receptor for S. S1: S1 domain of S. S2: S2 domain of S. RBD: receptor binding domain. NTD: N-terminal domain of S1. TMD: transmembrane domain. Structural configurations adapted from [45,46,34].

Preclinical vaccine development for both SARS-CoV and MERS-CoV has largely aimed to stimulate a robust immune response against the viral envelope-protruding spike (S) glycoprotein [28,29], a class I fusion protein, and/or the nucleocapsid (N) protein [30,31]. MERS-CoV S is proteolytically cleaved by host furin [32] during maturation into an S1 domain responsible for binding to DPP4 as well as an S2 domain containing two heptad-repeat regions that facilitate membrane fusion (Figure 1). The S1 domain can be further divided into the N-terminal domain (NTD), or S1^A^, associated with binding sialic acid [33], and the receptor binding domain (RBD), comprising the majority of the C-terminal domain of S1. Cryo-electron microscopy studies have shown that the RBD is flexible and opens upward or away from the viral envelope in order to establish contact with DPP4, which may expose S2’ [34], a second protease cleavage site within S2. Cleavage at S2’ is necessary for membrane fusion upon viral entry [32]. The centrality of S to viral entry helps explain why antibodies that target it are potently neutralizing [35]. On the other hand, while CoV N proteins are abundantly expressed during infection [36], immunization with SARS-CoV N did not induce strongly neutralizing antibodies [37], likely because N is not displayed on the viral surface. However, N is more conserved than S within CoV lineage [38], and vaccination with SARS-CoV N was shown to induce cytotoxic T cell responses in mice [39]. Therefore, N may help induce cell-mediated immunity to CoV infection [40], as may S [41]. CD4+ and CD8+ T cell responses from recovered MERS-CoV patients were particularly strong towards N peptides [42]. However, vaccination with N-based immunogens may carry risks associated with T_h_2-related eosinophilic immune enhancement [43], as may S-based vaccines [44]. Notwithstanding, because of the protection afforded by the robust immune response it generates, S has been the target of most vaccine candidates for MERS-CoV. In this review, we summarize the current state of MERS-CoV vaccine candidates and also describe potential barriers to MERS-CoV vaccine efficacy that first surfaced during research on developing a SARS-CoV vaccine.

## 2. Subunit Vaccines: Immunogenically Focused

Subunit vaccines comprise one or more immunogenic components derived from a pathogen [47]. They have gained popularity in recent decades due to the relative ease of their production and their reduced risks in vivo compared to vaccine types that involve live virus, namely live attenuated vaccines, viral vector vaccines, and even improperly prepared inactivated vaccines. 

### 2.1. Receptor Binding Domain

It is known from studies of recovered SARS-CoV patients that antibodies generated against the receptor binding domain (RBD) are both long-lasting (>3 years) and neutralizing [48]. The RBD in the MERS-CoV S glycoprotein was initially mapped to a region spanning residues 358 to 662 [49,50]; antisera from RBD protein-immunized mice or rabbits protected against in vitro infection with MERS-CoV. In further studies exploring immune correlates of protection, intranasal administration of RBD protein induced S1-specific immunoglobulin (Ig) G1, IgG2a, IgG3, lung IgA, and neutralizing antibodies (NAb, that is, untyped antibodies shown to functionally inhibit free virus from infecting cells) [51], as well as cell-mediated responses as measured by IL-2 and IFN-γ production in antigen-stimulated CD4+ and CD8+ splenocytes [52]. In this and subsequent studies, the RBD protein was fused to a fragment crystallizable (Fc) region of human IgG1 to increase the in vivo half-life of the immunogen [53]. Profiling of the immunogenic region of the RBD indicated residues 377-588 bound with the highest affinity to soluble DPP4 and induced the highest NAb titers, when administered to both mice and rabbits [54]. This range-refined RBD protein vaccine has been stably expressed in a modified high-yield CHO cell line [55]. Purified and adjuvanted with AddaVax^TM^ (MF59-like), the RBD protein vaccine was protective when administered intramuscularly to transgenic mice expressing human DPP4 (hDPP4), with no evidence of immunological toxicity or eosinophilic immune enhancement. 

Since the S glycoprotein exists in trimeric form on the virion, a quality lost in shortened forms of the protein, a trimer of this RBD protein vaccine has been generated containing a foldon trimerization motif [56]. This RBD protein trimer has been shown to elicit long-lasting NAb and be protective in challenged hDPP4-transgenic mice [57]. Independently, a monomeric RBD protein vaccine has been developed and tested in rhesus macaques, where it reduced MERS-associated lung pathology and reduced viral loads when adjuvanted with alum and administered intramuscularly in a three-dose regimen prior to challenge [58]. Finally, RBD proteins encoding sequences from different strains of MERS-CoV have been shown to induce cross-neutralizing antibodies against divergent human and camel MERS-CoV strains as well as monoclonal antibody (mAb) escape mutants, confirming the promise of the RBD as a valid vaccine target [59]. 

### 2.2. Full-Length S

Targeting the entire S glycoprotein has the advantage of including non-RBD neutralizing epitopes, including those in the more conserved domains. S protein “nanoparticles,” protein aggregates containing full-length S, have been proposed as a subunit vaccine, as nanoparticle vaccination adjuvanted with Matrix-M1^TM^ elicited NAb in mice [60] protected adenovirally hDPP4-transduced mice [24] from MERS-CoV challenge [61]. Measurements of viral titer and viral RNA were near the limit of detection in these vaccinated mice. However, despite these promising results, antibody-dependent enhancement (ADE) of infection was previously noted in the context of vaccination with full-length SARS-CoV S protein vaccine [62]. While ADE has not been demonstrated with full length S from MERS-CoV, further studies must consider this as a potential issue.

A variation of the full-length S glycoprotein vaccine is a trimer of S ectodomain (all but the transmembrane domain) conformationally locked in the prefusion state by the substitution of two proline residues in the S2 domain [34]. Work on related fusion proteins such as the F glycoprotein of respiratory syncytial virus has shown that stabilizing the glycoprotein in its prefusion state helps elicit a stronger neutralizing antibody response [63]. This “prefusion” S administered to mice elicited sera with greater neutralization activity against a panel of pseudoviruses bearing strain-specific variants of MERS-CoV S as compared to wild-type S. Since the S2 domain of CoVs is more conserved than the S1 domain [64], targeting epitopes in S2 may provide broader protection against different MERS-CoV strains and other lineage C βCoVs.

### 2.3. N-Terminal Domain

RBD- and full-length S-based protein vaccines build upon prior vaccine efforts for SARS-CoV. In contrast, vaccines targeting the N-terminal domain of S1 (NTD) offer a novel target. The NTD of S1 does not contain the RBD for MERS-CoV; however, the NTD binds sialic acid and is key to infecting certain cell types [33]. Immunization with NTD protein protected against MERS-CoV challenge in adenovirally hDPP4-transduced mice, inducing cell-mediated responses in splenocytes (CD8+ IFN-γ production, CD4+ IL-2 production, and IL-17A production) as well as humoral responses (IgG and NAb), although NAb titer was lower compared to that of an RBD protein vaccine [65]. Overall, these results suggest that targeting S1 domains outside the RBD may be a viable strategy for MERS-CoV vaccines.

## 3. DNA Vaccines: Efficient Protection

DNA vaccines offer a rapid platform to design and deliver immunogenic proteins, typically encoded on plasmid vectors and injected into tissue with accompanying electroporation [66]. DNA vaccine administration via electroporation has been tested in clinical trials with immunogenicity comparable to other vaccine types and predominantly low-grade adverse events reported [67]. The in vivo expression of plasmid-encoded proteins recapitulates native post-translational modifications while maintaining the capacity to stimulate both humoral and cell-mediated immunity [68]. While concerns about the safety of DNA vaccines and their potential to integrate into host cell chromosomes were voiced early in their development [69], integration using various plasmids and inserts appears to be extremely rare [70]. Several DNA vaccines for MERS-CoV have been reported to date.

### 3.1. Full-Length S

pVax1^TM^ is a proprietary, optimized plasmid vaccine vector that has been developed as a MERS-CoV vaccine by encoding a consensus MERS-CoV S glycoprotein containing codon and other proprietary optimizations, as well as an IgE leader sequence to promote expression and mRNA export [71]. Intramuscular administration of this construct with electroporation induced antibodies with cross-MERS-CoV-strain neutralization and antigen-specific, polyfunctional T cell responses in rhesus macaques. These humoral and cell-mediated immune responses correlated with minimal lung pathology and reduced lung viral loads upon MERS-CoV challenge. The same study reported NAb induction in dromedary camels, which indicates that the vaccine could be used in zoonotic reservoirs. Building upon these preclinical results, the pVax1^TM^ vaccine (GLS-5300) has completed a Phase I clinical trial (clinicaltrials.gov/ct2/show/NCT02670187).

A second vaccine platform utilizes pVRC8400 [72,73], a plasmid vector engineered for high transgene expression and enhanced cell-mediated responses. A vaccine regimen consisting of intramuscular administration of MERS-CoV strain England1 full-length S encoded on pVRC8400, with electroporation, and an AlPO_4_-adjuvanted S1 protein booster, induced NAb in rhesus macaques up to 10 weeks following booster [74]. This vaccine resulted in lower lung pathology upon challenge with the MERS-CoV strain JordanN3. In the same study, the full-length S DNA/S1 protein vaccine induced higher NAb titer in mice than other prime/boost combinations involving constructs encoding either S1 or S with the transmembrane domain (TMD) deleted. Consistent with this finding, the study reported that mAbs induced against domains outside the RBD were able to neutralize MERS-CoV pseudovirus. Together, these results reiterated the immunogenic potential of non-RBD epitopes including those derived from S quaternary (trimer) structure, which could help generate immune responses able to minimize escape variants derived from immunization targeting either the RBD or the S1 domain alone.

### 3.2. S1 Domain

The S1 domain from MERS-CoV strain Al-Hasa_15_2013 encoded on pcDNA^TM^3.1(+), a proprietary plasmid from which pVax1^TM^ is derived, has also been tested as a vaccine platform. This vaccine induced NAb in mice when given intramuscularly and antigen-specific cytokine production including CD4+ and CD8+ production of both IL-4 and IFN-γ in murine splenocytes [75]. In addition, the vaccine protected adenovirally hDPP4-transduced mice against challenge with MERS-CoV strain EMC/2012. Moreover, this S1 vaccine elicited more NAb than did full-length S in the same vector. This result was attributed to increased secretion of the S1 protein, which lacked a TMD, and greater uptake by antigen-presenting cells [76]. A separate group’s study comparing full-length S- and S1-encoding pcDNA^TM^3.1 vaccines found that the S1 vaccine elicited a more balanced IgG2a/IgG1 ratio in mice compared to that elicited by full-length S [77] suggesting a balanced T_h_1/T_h_2 response [78]. Together, these results show that multiple plasmid vaccine vectors encoding either full-length S or the S1 domain induce adaptive immunity and protect against MERS-CoV challenge.

## 4. Viral Vector Vaccines: Optimized Delivery

Viral vector vaccines contain one or more immunogenic proteins of the pathogen of interest in the context of an attenuated virus backbone. This approach takes advantage of cellular entry by the virus as well as adjuvantation from viral components, and induces both humoral and strong cell-mediated responses [79]. Early studies into viral vector vaccines for MERS-CoV built upon established platforms and have subsequently transitioned to newer viral vector approaches.

Venezuelan equine encephalitis (VEE) virus replicon particles (VRPs), an alphavirus-based platform that replaces the VEE structural genes with a foreign transgene, has been shown to induce strong humoral and cellular immune responses [80,81]. A VRP encoding MERS-CoV S elicited NAb in both young and aged mice [38]. Additional studies with this vector have shown that immunization with an N protein-expressing VRP protected adenovirally hDPP4-transduced mice from MERS-CoV challenge in a CD4+ T cell- and IFN-γ-dependent manner [82]. Moreover, a specific N protein epitope was stimulatory in mice transgenic for human leukocyte antigen DR2 and DR3, highlighting the relevance of this epitope to human antigen recognition and to promoting cell-mediated immunity in humans.

Modified vaccinia virus Ankara (MVA) [83,84], a well-established vaccine platform, has been developed to encode full-length MERS-CoV S. This vaccine induced NAb and CD8+ T cell responses in mice [85] and also protected against MERS-CoV-induced histopathology in adenovirally hDPP4-transduced mice before challenge [86]. Moreover, minimal inflammation and lymph node hyperplasia was observed at the site of injection [87]. This same MVA-MERS-CoV S vaccine injected intramuscularly into dromedary camels was shown to induce NAb and to limit excretion of infectious virus upon intranasal challenge with MERS-CoV [88]. A Phase I clinical trial is underway (clinicaltrials.gov/ct2/show/NCT03615911).

Adenoviruses compose a third platform of viral vectors for MERS-CoV vaccines. Adenovirus-vectored vaccines have been tested in clinical trials for a wide variety of diseases, notably HIV [89]. However, their efficacy may be hampered by pre-existing immunity to prevalent adenovirus serotypes [90,91]. For example, pre-existing immunity to human adenovirus serotype 5 (Ad5) was shown to result in reduced CD8+ T cell responses against an Ad5-vectored transgene [92]. To take advantage of the shared respiratory route of infection of both MERS-CoV and adenovirus, Ad5-vectored full-length S and S1 vaccines have been developed [93]. These elicited antigen-specific IgG and NAb when administered intramuscularly to mice with subsequent intranasal boosting. Importantly, this study did not detect immunity against the Ad5 vector in dromedary camels, the intended vaccination population. Moreover, camel peripheral blood mononuclear cells and a camel-derived fibroblast cell line were able to be infected with Ad5. Another Ad5-MERS-CoV S vaccine has been separately developed, as has a human adenovirus type 41 (Ad41)-MERS-CoV S vaccine [94]. Adenovirus type 41 (Ad41) is an enteric pathogen with potential use as an orally administered vaccine [95]. Both of these vaccines, Ad5-MERS-CoV S and Ad41-MERS-CoV S, were reported to induce humoral responses when administered intragastrically in mice. In addition to humoral responses, they also induced long-lasting cell-mediated responses in the lung and spleen when administered intramuscularly. One final Ad5-based MERS-CoV immunization regimen has been reported [96]. Immunizing with Ad5-vectored S followed by boosting with S nanoparticles induced S-specific IgG, NAb, and both T_h_1 and T_h_2 cell-mediated responses in mice, and also protected adenovirally hDPP4-transduced mice from MERS-CoV challenge.

To circumvent the seroprevalence of circulating human adenoviruses, chimpanzee adenoviruses have also been developed as viral vaccine vectors [97] and have entered clinical trials [98]. A MERS-CoV S-encoding vaccine based on a chimpanzee adenoviral vector (ChAdOx1) was shown to induce high levels of NAb and cell-mediated responses (CD8+ IFN-γ, TNFα, and IL-17 production) in mice 4 weeks post-immunization [99]. This vaccine was constructed with a codon-optimized S glycoprotein sequence and the tissue plasminogen activator (tPA) gene leader sequence to promote secretion [100,101]. The ChAdOx1-MERS-CoV S vaccine protected against lethal challenge in a transgenic hDPP4 mouse model [102]. Based on previous work with the ChAdOx1 vector demonstrating its safety in humans, the ChAdOx1-MERS-CoV S vaccine is undergoing a Phase I clinical trial (clinicaltrials.gov/ct2/show/NCT03399578).

Several additional viral vectors have been employed as MERS-CoV vaccines. Measles virus vector platforms have been developed over the past two decades [103]. A full-length or soluble form of S encoded in measles vaccine strain MV_vac2_ induced NAb, proliferation of T cells, S-specific IFN-γ production, and cytotoxic activity [104]. The vaccine also protected against MERS-CoV challenge in adenovirally hDPP4-transduced mice that were transgenic for a measles virus receptor. Further characterization of the T cell responses induced by this vaccine has been performed [105]. Of note, 5-fold higher numbers of reactive T cells were induced by vaccination with MV_vac2_-S than those induced by N protein using the same vector. Additionally, antigen-specific IFN-γ production by T cells could be induced in older mice (7 months old) at levels near those induced in younger mice (6–12 weeks old). 

Newcastle disease virus (NDV) has been explored as a vaccine vector as it infects the respiratory tract and can induce systemic and mucosal immunity in non-human primates [106]. An NVD vector expressing MERS-CoV S was shown to induce long-lasting (up 14 weeks post-immunization) NAb titers in camels [107]. The research group behind this study also examined vesicular stomatitis virus (VSV) [108] as a viral vector. S expressed from a VSV reverse genetics system was shown to incorporate onto the surface of virions rescued in cell culture. Purified vaccine was able to infect cells in an hDPP4-dependent manner, induced S-specific IgG and NAb in mice, and stimulated humoral and cell-mediated (IFN-γ-production) responses in rhesus macaques [109]. 

Similar to the VSV platform, a rabies virus (RABV) vector has been explored. Inspired by studies combining rabies and Ebola vaccine platforms [110], a β-propiolactone-inactivated dual rabies/MERS vaccine has been proposed which incorporates the MERS-CoV S1 domain fused to rabies virus G protein on the RABV virion [111]. This vaccine elicited S-specific IgG and NAb and fully protected adenovirally hDPP4-transduced mice from MERS-CoV challenge. The VSV and RABV approaches described here are unique in that they encode S (or S1) in the vector genome and also display it on the virion surface.

Finally, virus-like particles (VLPs), which comprise self-assembling immunogenic proteins, but no genome [112], have also been used as viral vectors. A baculovirus VLP containing S as well as MERS-CoV envelope and matrix proteins elicited RBD-specific IgG and IFN-γ responses in rhesus macaques [113]. A subsequent baculovirus VLP vaccine was developed that focused only on a fusion of the RBD from S and the immunogenic VP2 protein of canine parvovirus. This vaccine induced RBD-specific IgG, NAb, and cell-mediated responses including IFN-γ, IL-2, and IL-4 production in mice, and also activated dendritic cells in inguinal lymph nodes [114]. In summary, a variety of viral vector vaccines for MERS-CoV induce promising immune responses in animal models and often demonstrate protection from challenge.

## 5. Live Attenuated and Inactivated Vaccines: Situationally Useful

A final approach to developing MERS-CoV vaccines delivers the whole virus, either inactivated or live but attenuated. Both of these vaccine types resemble the original virus, preserving structural features and a full or nearly-full repertoire of immunogenic components. Inactivated viruses may contain structural deformations introduced by inactivation, but, unlike attenuated viruses, they pose no risks, if properly inactivated, either of reversion to a virulent state or persistent infection in immunocompromised patients. Fewer examples of whole virus vaccines, compared to the other vaccine types, have been developed for MERS-CoV.

### 5.1. Inactivated

Development of inactivated vaccines for MERS-CoV has been stymied by prior concerns with SARS-CoV inactivated vaccines. Eosinophil-related lung pathology was observed for a SARS-CoV vaccine doubly inactivated with both formalin and UV irradiation [115]. This response was particularly notable in aged mice versus young mice, and following heterologous versus homologous challenge. Similarly, immunization with a gamma-irradiated MERS-CoV vaccine adjuvanted with either alum or MF59 elicited NAb and reduced viral titer upon challenge in hDPP4-transgenic mice, but induced eosinophil-related lung pathology in vaccinated mice after challenge [116].

A different inactivation method was tried for a second MERS-CoV inactivated vaccine. Formalin-inactivated MERS-CoV adjuvanted with alum and oligodeoxynucleotides containing unmethylated CpG motifs was shown to elicit levels of NAb on par with those elicited by an S glycoprotein-only vaccine [117]. Moreover, the vaccine offered better protection than S alone based on reduction of lung viral titer in adenovirally hDPP4-transduced mice after MERS-CoV challenge. Remarkably, eosinophil-mediated vaccine-related pathology was not observed in this animal model. Interestingly, it has also been shown that including Toll-like receptor agonists in a UV-inactivated SARS-CoV vaccine reduced T_h_2-associated pathology in lungs after challenge [118]. These results suggest that inactivated CoV vaccines may remain viable options for further development with the right inactivation method and adjuvants.

### 5.2. Live Attenuated

Live attenuated vaccines for MERS-CoV show efficacy in animal models, but so far have not been pursued in subsequent studies. While riskier than other vaccine types, live attenuated vaccines have historically offered protection against a variety of threatening illnesses [119] and may be reserved for outbreak scenarios where they offer an immunogenically robust solution. The CoV envelope (E) protein is important in virion assembly and egress, and has also been shown to inhibit the host cell stress response [120]. An E-deletion mutant of SARS-CoV was previously found to be protective in vivo against SARS-CoV challenge [121]. An initial study into a MERS-CoV reverse genetics system reported on a replication-competent, but propagation-defective mutant lacking the E protein that could be rescued in cell culture with E expressed in *trans* [122]. However, the E-deletion mutant was rescued at 100-fold lower titer compared to wild-type MERS-CoV, perhaps explaining why this mutant has not been further developed as a live attenuated vaccine candidate. 

Other CoV components have been targeted in live attenuated vaccine development. Nonstructural protein 14 (nsp14) contains an exoribonuclease (ExoN) essential to replication fidelity that is found in all known nidoviruses with genome sizes greater than 20 kb [5]. A stable deficiency in nsp14 attenuated SARS-CoV in young, aged, and immunocompromised mice, and was able to induce protection following vaccination [123]. However, ExoN mutants for MERS-CoV have not been reported.

CoV gene nonstructural protein 16 (nsp16) is a 2′-O-methyl-transferase involved in viral mRNA capping [124] and was previously inactivated in a SARS-CoV live attenuated vaccine [125,126]. A live nsp16-deficient MERS-CoV vaccine was similarly attenuated in a type I interferon- and IFIT1-dependent manner. Immunization with the MERS-CoV nsp16 mutant induced NAb and protected CRISPR-engineered hDPP4-transgenic mice [23] from challenge with a mouse-adapted MERS-CoV strain [127]. 

Finally, live attenuated vaccines lacking CoV accessory proteins have also been considered. CoV accessory proteins are dispensable for viral replication but have been shown to modulate interferon signaling and pro-inflammatory cytokine production [128]. A MERS-CoV strain lacking accessory open reading frames (ORFs) 3, 4, and 5 was attenuated in vivo, induced NAb, and like the nsp16 mutant, protected CRISPR-engineered hDPP4-transgenic mice from challenge with a mouse-adapted MERS-CoV strain [129]. Overall, whole vaccine approaches to MERS-CoV vaccination appear both protective and safe in animal models.

## 6. Conclusions and Future Directions

Aided by knowledge gained from vaccine development against SARS-CoV and other contemporary viral diseases, MERS-CoV vaccine development efforts have multiplied since its emergence, yielding promising vaccine candidates spanning multiple platforms (Table 1). Nevertheless, key barriers to vaccine efficacy first noted for SARS-CoV may also hold true for MERS-CoV. As with SARS-CoV [130], mortality from MERS-CoV has disproportionately affected the aged. Additionally, immunocompromised individuals and those with chronic conditions are at greater risk of mortality from MERS-CoV infection [11]. A universal MERS-CoV vaccine must offer protection to these vulnerable classes of people. 

More studies on the effectiveness of the proposed MERS-CoV vaccines in models of immunosenescence, immunocompromise, and chronic conditions are needed. In this regard, vaccination studies with SARS-CoV have indicated that vaccines may be capable of inducing protection in young animals while failing to protect aged animals [115]. In light of the threat of related coronavirus strains emerging, MERS-CoV vaccine studies must also consider heterologous challenge models to ensure safety from vaccine-induced immunopathology, especially in older individuals [115]. In short, vaccine-induced immunopathology, especially T_h_2-related eosinophilic immune enhancement [131], from both homologous and heterologous challenge should be specifically monitored in vulnerable populations. Interestingly, it was shown that pathology resulting from MERS-CoV challenge in the lungs of immunosuppressed rhesus macaques was lower compared to that of non-immunosuppressed macaques, underscoring the immunopathogenic component of respiratory disease caused by CoVs [132]. 

The different vaccine platforms described herein have unique advantages and disadvantages. Since severe CoV disease maintains an immunopathogenic component, a successful vaccine must strike a balance between protection and excessive immune activation. As seen with full-length S [44] as well as inactivated virus [116], vaccination may produce immunopathology under certain conditions. Alternatively, protection must be thorough enough to prevent NAb escape, a phenomenon inversely correlated with the number of immunogenic epitopes. While antibodies induced against S of either SARS-CoV or MERS-CoV poorly cross-neutralize across their respective lineages [38], a vaccine that contains multiple immunogenic epitopes would perhaps also offer greater cross-protection against heterologous strains within lineage as they emerge, especially if conserved epitopes are included in vaccine design. A greater understanding of MERS-CoV pathology will also help guide future vaccine development efforts by illuminating possibly critical differences in vaccine responses between MERS-CoV and SARS-CoV, the latter of which has largely influenced vaccine development against the former.

Overall, MERS-CoV vaccines have shown encouraging results in preclinical studies and we hope these vaccines stand up to safety considerations in order to proceed through clinical trials. While development of therapeutic treatment is critical, vaccination carries the promise of mitigating future outbreaks and alleviating disease burden from the most vulnerable populations including the aged, the immunosuppressed, healthcare workers, family members of infected patients, and those in endemic areas.

## Figures and Tables

**Table 1:** MERS-CoV vaccine candidates grouped according to category.

Vaccine Type	Humoral Response in:	Cell-Mediated Response in:	Protective in:	Clinical Trial	Source (s)
**Subunit**					
RBD	M, P	M, P	M, P		[50,55,58]
S nanoparticles	M		M		[61]
Prefusion-locked S	M				[34]
NTD	M	M	M		[65]
					
**DNA**					
pVax1-S	M, P, C	M, P	P	Phase I	[71]
pVRC8400-S ^1^	M, P		P		[74]
pcDNA3.1(+)-S1 or S	M	M	M		[75,77]
					
**Viral Vector**					
VEEV-S	M				[38]
VEEV-N		M	M		[82]
MVA-S	M, C	M	M, C	Phase I	[88]
Ad5-S or S1	M	M			[93,94]
Ad5-S ^2^	M	M	M		[96]
Ad41-S	M	M			[94]
ChAdOx1-S	M	M	M	Phase I	[102]
MVvac2-S	M	M	M		[104]
Newcastle-S	M, C				[107]
VSV-S	M, P	P			[109]
Rabies-S1	M		M		[111]
Bac-S,E,M	P	P			[113]
Bac-RBD+VP2	M	M			[114]
					
**Whole**					
Formalin inactivated	M		M		[117]
MERS-ΔE					[122]
MERS-dNSP16	M		M		[127]
MERS-dORF3-5	M		M		[129]

Humoral response denotes any antibody response generated, in most cases a NAb response. Cell-mediated responses denote T cell activation markers including IFN-γ. S: MERS-CoV spike protein. N: MERS-CoV nucleocapsid. RBD: receptor binding domain. NTD: N-terminal domain. S1: spike subdomain S1. Bac: baculovirus VLP. E: MERS-CoV envelope protein. M (under *Vaccine Type*): MERS-CoV membrane protein. VP2: canine parvovirus VP2 protein. M (under *Protective in:*): mouse. P: non-human primate. C: camel. ^1^ With S1 protein booster; ^2^ with S nanoparticles booster.
